# 2280. Surveillance of Life-Long Antibiotics (SOLLA) 2: Patients on Life-Long Antibiotics in an Australian Healthcare Network

**DOI:** 10.1093/ofid/ofad500.1902

**Published:** 2023-11-27

**Authors:** Taylah van Leerdam, Jillian Lau, Ian Woolley, Mitchell Browne, Jessica O’Bryan

**Affiliations:** Monash Health, Melbourne, Victoria, Australia; Alfred Health, Melbourne, Victoria, Australia; Monash Health, Melbourne, Victoria, Australia; Monash Health, Melbourne, Victoria, Australia; Monash Health, Melbourne, Victoria, Australia

## Abstract

**Background:**

There is a range of conditions for which long-term antibiotics are prescribed, most of which lack high quality evidence. Apart from promoting antimicrobial resistance, long-term antibiotics also impact the individual patient, disrupting the normal microbiome, and increasing the risk of infection with multi-resistant organisms (MROs). Previous work conducted at Monash Health, in Victoria, Australia, in 2014 noted great heterogeneity in both indications for prolonged antibiotic courses and the antimicrobial agents used.

**Methods:**

The current study aimed to reassess prescribing trends within the same health network, across two time periods in 2019 and 2021. Outpatient prescriptions were extracted from the Monash Health pharmaceutical database at two time points, from 1^st^ January 2019 – 30^th^ June 2019, and 1^st^ January 2021 – 30^th^ June 2021.

**Results:**

From 4,704 outpatient prescriptions, 929 patients were identified as being on either one or more antibiotics for an intended duration of ≥ 12 months. There were a total 536 (57.7%) males and 393 females (42.3%), with a mean age of 58.20 years. Sixty-eight patients died between the two follow-up periods. Data was separated into 3 groups: primary prophylaxis in the setting of immunosuppression (n = 687), secondary prophylaxis for infections deemed incurable (n = 78), and “Other” (n = 164). Six hundred and four patients (88%) on primary prophylaxis were for *Pneumocystis jirovecii* pneumonia, and 83 (12%) post splenectomy. Trimethoprim/sulfamethoxazole was predominantly prescribed for PJP prophylaxis (n = 583), followed by dapsone (n = 21). Prosthetic joint infections made up 57 (73%) of those on suppressive antibiotics for infections deemed incurable. An array of antibiotics was utilised to treat them, as seen in Figure 1. The “Other” category included those on antibiotics for difficult to treat infections, such as mycobacterial infections (n = 43), chronic airways disease (n = 38), spontaneous bacterial peritonitis (n = 23) and recurrent urinary tract infections (n = 12). Data on comorbidities, hospitalisations and MROs between groups can be viewed in Table 1.

**Figure:**
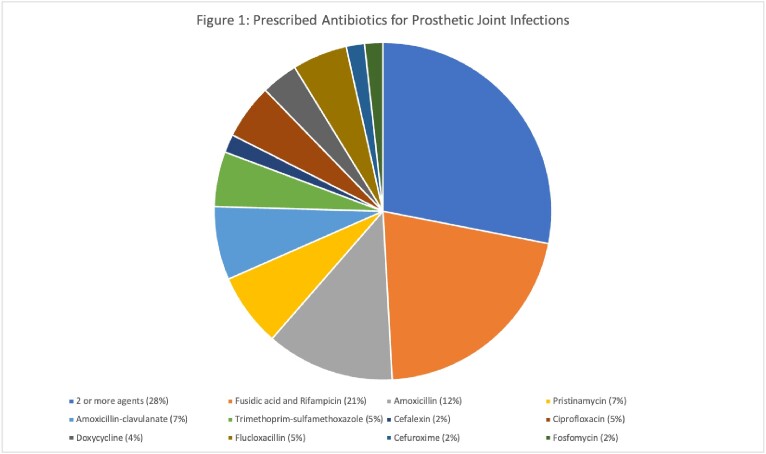

**Figure:**
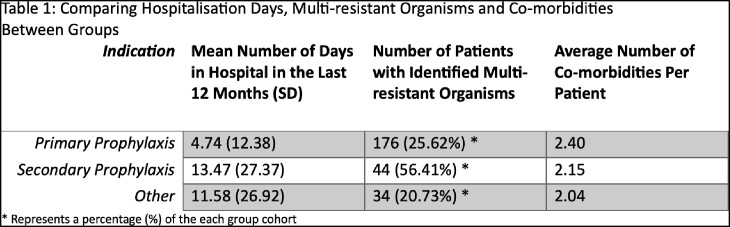

**Conclusion:**

Further research is needed. Prospective data examining long-term patient outcomes, and patterns of prescribing across different health networks would be of benefit.

**Disclosures:**

**Jillian Lau, MBBS, FRACP**, Gilead: Advisor/Consultant|Gilead: Grant/Research Support|MSD: Grant/Research Support|Viiv Healthcare: Advisor/Consultant **Ian Woolley, MBBS, FRACP, DTM&H, MD**, Gilead: Educational support, investigator for Gilead supported trial|MSD: educational support, participation in a clinical trial|Pfizer: Educational support|ViiV: Educational Programs, Advisory Board member, ViiV sponsored trials

